# The pro-fibrotic role of autophagy in renal intrinsic cells: mechanisms and therapeutic potential in chronic kidney disease

**DOI:** 10.3389/fcell.2024.1499457

**Published:** 2024-12-11

**Authors:** Ying-Ying Zhang, Xiao-Tao Zhou, Geng-Zhen Huang, Wen-Jun Liao, Xian Chen, Yue-Rong Ma

**Affiliations:** ^1^ School of Basic Medicine, Chengdu University of Traditional Chinese Medicine, Chengdu, China; ^2^ The Affiliated Hospital of Southwest Medical University, Luzhou, China; ^3^ Hospital of Chengdu University of Traditional Chinese Medicine, Chengdu, Sichuan, China; ^4^ Chengdu second people’s Hospital, Chengdu, China

**Keywords:** chronic kidney disease, renal fibrosis, autophagy, mechanism, therapeutic targets

## Abstract

Chronic kidney disease (CKD) represents a significant global public health burden, affecting over 10% of the world’s population. Its high morbidity, multifactorial complications, and substantial mortality impose significant burdens on healthcare systems and patients, necessitating considerable investment in healthcare resources. Renal fibrosis (RF) is a key pathological feature and driver of CKD progression. Extensive research indicates that autophagy participates in the complete pathogenesis of RF. Under physiological conditions, autophagy is essential for maintaining renal cellular homeostasis. However, under pathological conditions, perhaps aberrant and sustained activation of autophagy contributes to oxidative stress, apoptosis, inflammation, etc. Ultimately, they accelerate the development of RF. The role of autophagy in RF is currently controversial. This review investigates the molecular mechanisms by which intrinsic renal cell autophagy contributes to RF across diverse disease models, suggesting that autophagy and its associated regulatory pathways represent potential diagnostic and therapeutic targets for CKD.

## 1 Introduction

Chronic kidney disease (CKD) is a progressive disorder characterized by chronic structural and functional damage to the kidneys, with diverse etiologies. It is associated with high morbidity, significant mortality, and a range of debilitating complications (Drawz and Rahman, 2015). The latest Global Burden of Disease (GBD) study reports a global prevalence of CKD exceeding 10%, with a continuing upward trend. By 2040, CKD is projected to be the fifth leading cause of global mortality, imposing substantial burdens on healthcare systems and patients, demanding significant healthcare resource allocation (Kalantar-Zadeh et al., 2021). Importantly, renal fibrosis (RF) is a key pathological feature and driver of CKD progression. Severe RF is frequently observed in patients with CKD progressing to end-stage kidney disease (ESKD) (Liu, 2011). Elucidating the molecular mechanisms underlying RF and developing effective interventions are, therefore, crucial for advancing CKD research.

RF results from a dysregulated tissue repair response triggered by various factors, including trauma, metabolic disorders, chronic inflammation, and autoimmune processes. In kidney disease, this dysregulated repair process results in excessive extracellular matrix (ECM) deposition and scar formation. This process involves the sustained activation and expression of key pro-fibrotic cytokines, including transforming growth factor-beta 1 (TGF-β1), connective tissue growth factor (CTGF), tumor necrosis factor-alpha (TNF-α), interleukin-6 (IL-6), alpha-smooth muscle actin (α-SMA), and collagen type I (Col-I). This is accompanied by epithelial–mesenchymal transition (EMT), increased mesenchymal cell activation, and excessive ECM deposition, ultimately disrupting the normal architecture of the renal tubules, glomeruli, and interstitium (Henderson et al., 2020).

**FIGURE 1:**
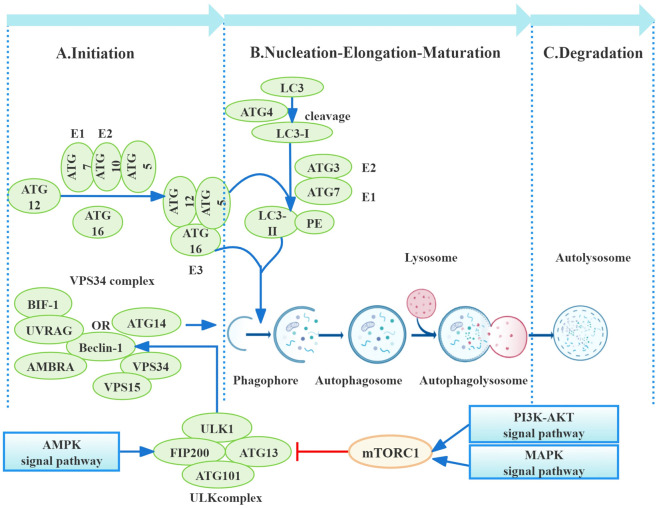
Process of autophagy. Autophagy, a multi-step process, is primarily regulated by autophagy-related proteins (ATGs). Initiation involves mTORC1 downregulation, leading to ULK1 and ATG13 dephosphorylation and subsequent autophagosome nucleation, influenced by pathways including PI3K/AKT, AMPK, and MAPK. Nucleation utilizes the VPS34 complex (beclin-1, VPS34, VPS15, and ATG14), further regulated by UVRAG, Bif-1, and AMBRA1. Elongation depends on ATG conjugation systems (ATG12–ATG5–ATG16 and LC3-PE), culminating in autophagosome maturation through microtubule-mediated transport to lysosomes for fusion and autolysosome formation. Finally, autolysosomal degradation completes the process (Galluzzi et al., 2017b).

Autophagy is a cellular process involving the degradation of damaged organelles and macromolecules in eukaryotic cells (Klionsky, 2007). Three main types of autophagy are distinguished based on the mechanism of substrate delivery to the lysosome: chaperone-mediated autophagy (CMA), micro-autophagy, and macro-autophagy. Macro-autophagy is the predominant form of autophagy (Galluzzi et al., 2017a). For the remainder of this review, the term “autophagy” will refer specifically to macro-autophagy. Autophagy is primarily initiated by autophagy-related (ATG) genes and modulated by various signaling pathways. Key regulatory pathways include the mammalian target of rapamycin (mTOR), mitogen-activated protein kinase (MAPK), AMP-activated protein kinase (AMPK), and phosphatidylinositol 3-kinase (PI3K)/protein kinase B (Akt) signaling pathways (Shu et al., 2023) (Figure 1). Recent research has revealed a complex interplay between ribonucleotide reductase (RRM2) and autophagy. RRM2 activation disrupts dNTP homeostasis (Wang et al., 2022), potentially inhibiting autophagy; however, receptor-mediated selective autophagy can degrade RRM2 transcripts (Tan et al., 2023). Furthermore, the limited research on the role of RRM2 in RF suggests this as a promising area for future investigation. Extensive research supports the multifaceted role for autophagy throughout the progression of RF (Kaushal et al., 2020; Tang et al., 2020). Autophagy is essential for maintaining renal cell homeostasis under physiological conditions. However, under pathological conditions, perhaps aberrant and sustained activation of autophagy contributes to a complex interplay of events, including apoptosis, oxidative stress, inflammation, pro-fibrotic factor secretion, cellular senescence, cell cycle arrest, and tissue damage, ultimately leading to RF (Galluzzi et al., 2017b; Beaulaton and Lockshin, 1977; Berry and Baehrecke, 2007; Yu et al., 2004; Ruby et al., 2023). As noted by Cybulsky (2017), protein misfolding and endoplasmic reticulum (ER) stress in RF are closely coordinated with autophagy. A recent review highlights dysregulation of FUNDC1-mediated mitophagy as a key contributor to the progression of various renal diseases, including RF (Li J. et al., 2024). Moreover, a recent study demonstrated that autophagy in renal tubular epithelial cells regulates fibroblast growth factor 2 (FGF2) expression via the extracellular signal-regulated kinase (ERK)/MAPK pathway, promoting fibroblast proliferation and fibrosis (Livingston et al., 2024). However, the mechanisms by which autophagy influences RF are complex and not yet fully understood. This review aims to elucidate the molecular mechanisms governing autophagy initiation and maintenance across various renal cell types, clarifying its pro-fibrotic role. Furthermore, we will discuss the potential of targeting autophagy for the detection, diagnosis, and treatment of renal diseases, aiming to inform efficient strategies for prevention and delay of CKD progression.

## 2 The regulatory mechanism and significance of autophagy in different cells in RF

### 2.1 Podocyte autophagy and RF

**FIGURE 2:**
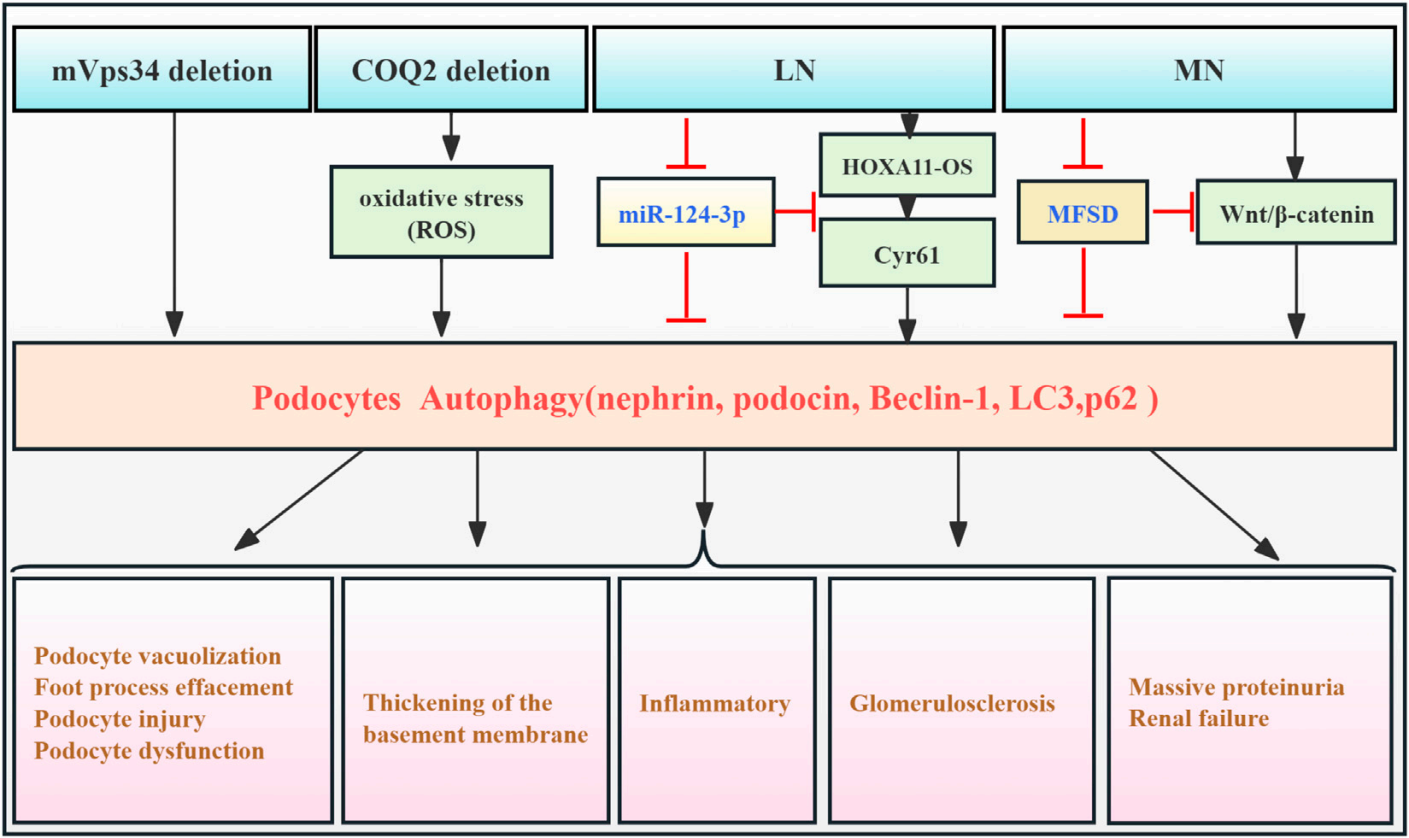
Role of podocyte autophagy in RF. Agents shown in black font exacerbate renal injury by promoting podocyte autophagy, whereas agents shown in blue font protect renal tissues by inhibiting autophagy and its associated regulators. Black arrows indicate promotion, and red “T”-shaped bars indicate inhibition. These studies demonstrate the deleterious effects of podocyte autophagy activation on the kidney. mVps34, the mammalian homolog of yeast vacuolar protein sorting defective 34; HOXA11-OS, the opposite strand of homeobox A11; Cyr61, cysteine-rich 61; MFSD, Mahuang Fuzi and Shenzhuo decoction; MN, membranous nephropathy; LN, lupus nephritis.

Podocytes, located in the outer layer of the glomerular filtration barrier (GFB), are crucial for glomerular filtration. RF causes podocyte damage and loss, disrupting the glomerular filtration barrier and leading to significant proteinuria (Asanuma et al., 2003). Extensive research indicates that podocytes exhibit the highest basal autophagy levels among intrinsic renal cells, playing a particular role in maintaining podocyte ultra-structure and GFB homeostasis (Pavenstädt et al., 2003). However, accumulating evidence suggests that elevated podocyte autophagy exacerbates podocyte injury in the context of RF. One study using a mouse podocyte model with silenced mVps34 demonstrated podocyte vacuolation, enlarged autophagosomes and autophagic lysosomes, and foot process effacement at 6 weeks. Glomerular sclerosis and RF were observed, accompanied by significant proteinuria and renal dysfunction. Significant increases in lysosomal markers (LAMP1 and LAMP2) and autophagosome markers (LC3-II/I) were detected in glomerular lysates. These findings suggested that mVps34 deletion leads to upregulation of autophagy, contributing to podocyte damage and RF. This emphasizes mVps34’s crucial function in regulating intracellular vesicle trafficking through the autophagic pathway (Chen et al., 2013). Silencing the COQ2 gene in a *Drosophila* nephropathy model induces oxidative stress, triggering mitophagy and macro-autophagy in podocytes. This finding established a link between COQ2 and the autophagy pathway and demonstrated that podocyte damage in this model is accompanied by aberrant autophagy activation (Zhu et al., 2017). Pan et al. (2022) observed upregulation of HOXA11-OS and downregulation of miR-124-3p in podocytes from the MRL/lpr model. This, in turn, resulted in upregulated Cyr61 and autophagy factors, ultimately contributing to cellular damage and exacerbating lupus nephritis (LN) progression. Additionally, Gao et al. (2022) showed that Mahuang Fuzi and Shenzhuo decoction (MFSD) mitigates podocyte damage in membranous nephropathy (MN) by suppressing the Wnt/β-catenin signaling pathway and, consequently, downregulating autophagy. This resulted in reduced RF. These pieces of evidence demonstrate that aberrant podocyte autophagy can be detrimental in kidney disease, accelerating disease progression. However, this contrasts with the findings of many studies, which suggest a protective role for podocyte autophagy in various kidney disease models (Barutta et al., 2023; Guo et al., 2024; Njeim et al., 2024). Further research is needed to fully clarify the role of podocyte autophagy in fibrosis development (Figure 2; Table 1).

**TABLE 1:** Factors influencing podocyte autophagy changes in RF.

Model	Autophagy-targeted cells	Drug/agent	Effect on autophagy	Mechanism and effect	References
mVps34pdKOmice	Podocytes	mVps34-knockout	↑ Autophagy	↑ LAMP1 and LAMP2 and LC3-II/I	Chen et al. (2013)
				↑ Podocyte damage and glomerulosclerosis	
COQ2 deletion *drosophila*	Podocytes	COQ2 deletion	↑ Autophagy	↑ Oxidative stress (ROS)	Zhu et al. (2017)
				↑ Mitochondrial and podocyte damage	
LN patients	Podocytes	HOXA11-OS	↑ Autophagy	↓ miR-124-3p and ↑ Cyr61 to activate autophagy	Pan et al. (2022)
MRL/lpr mice				↑ Podocyte damage and the progression of LN	
PHN rats	Podocytes	MFSD	↓ Autophagy	↓ Wnt/β-catenin signaling pathway	Gao et al. (2022)
				↓ Podocyte damage and the progression of MN.	

### 2.2 Endothelial cell autophagy and RF

The kidney contains a diverse population of endothelial cells (ECs), including glomerular ECs (GECs), vascular ECs (VECs), and lymphatic ECs (LECs). They reside in distinct renal microenvironments and perform specialized transport functions (Jourde-Chiche et al., 2019). GECs located in the inner layer of the GFB are particularly important for maintaining GFB integrity and supporting podocyte function. Renal endothelial dysfunction is a key contributor to the progression of CKD and RF (Rabelink and de Zeeuw, 2015). While the precise mechanisms underlying this dysfunction remain largely unclear, the role of autophagy in renal ECs remains largely unexplored. High glucose exposure in cultured mouse GECs inhibits AMPK signaling, upregulates autophagy, and promotes ECM remodeling. Conversely, exogenous H2S activates the STRAD/MO25/LKB1 pathway, leading to AMPK phosphorylation, autophagy inhibition, and reduced ECM deposition, thus mitigating GEC damage and preventing fibrosis (Kundu et al., 2013). Notably, Hu et al. (2017) developed sialic acid–polyethylene glycol–dexamethasone (SA-PEG-DXM) micelles, which accumulated in the kidneys of AKI mouse models, effectively inhibiting VEC autophagy (beclin-1, Atg5, and Atg12) and reducing pro-inflammatory cytokine production, thus mitigating kidney injury.

**FIGURE 3:**
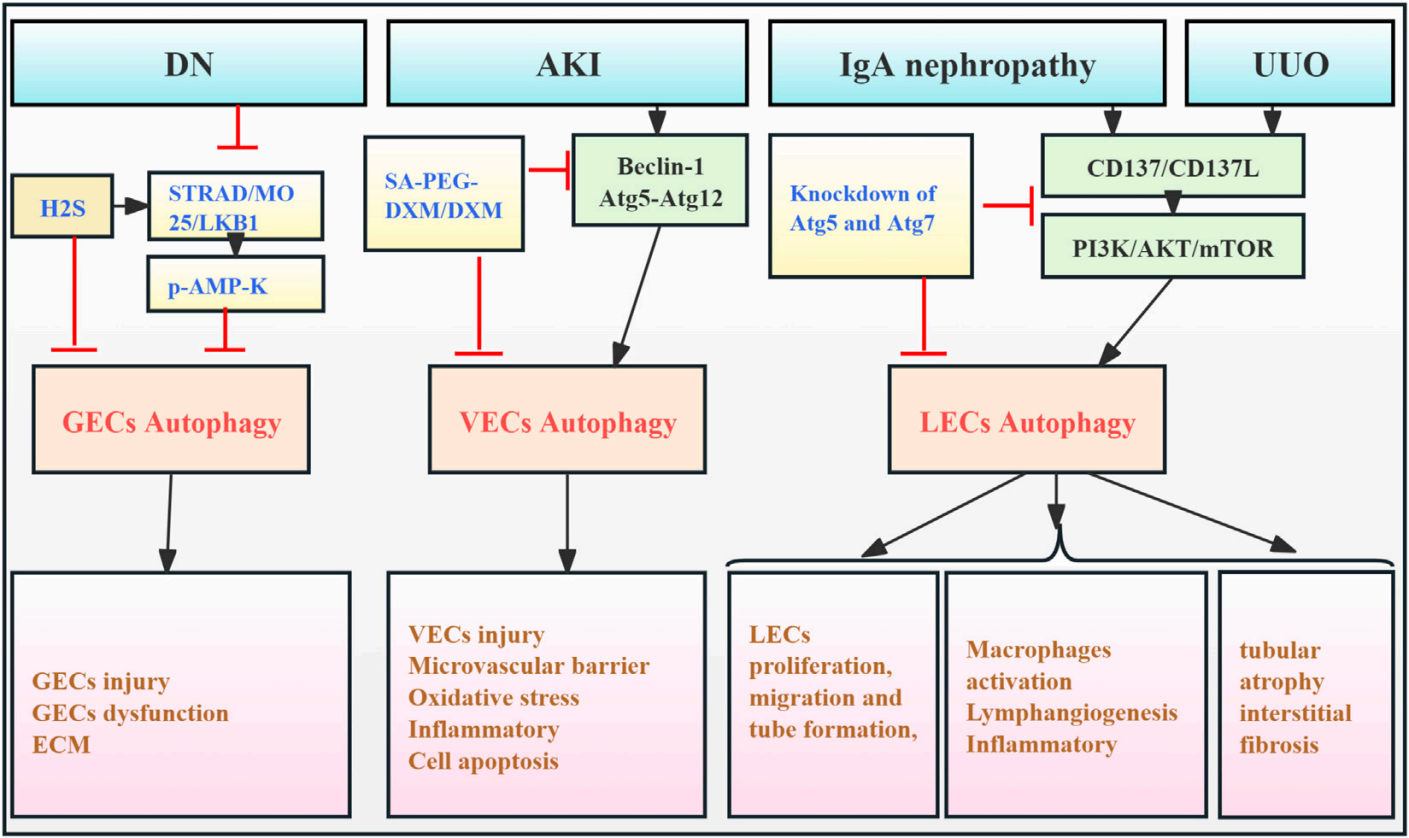
Role of EC autophagy in RF. Agents shown in black font exacerbate renal injury by promoting podocyte autophagy, whereas agents shown in blue font reduce renal tissues by inhibiting autophagy and its associated regulators. Black arrows indicate promotion, and red “T”-shaped bars indicate inhibition. These studies demonstrate the deleterious effects of EC autophagy activation on the kidney. ECs, endothelial cells; GECs, glomerular ECs; VECs, vascular ECs; LECs, lymphatic ECs; H2S, hydrogen sulfide; STRAD, STE- 20-related protein; MO25, mouse protein-25; LKB1, liver kinase B1; AMP-K, adenosine monophosphate-activated protein kinase; ECM, extracellular matrix; SA-PEG-DXM/DXM, sialic acid–polyethylene glycol–dexamethasone/dexamethasone.

In addition, another finding suggests that sustained VEC autophagy activation and inflammatory response in sepsis-related AKI models contribute to exacerbated tissue damage, poor prognosis, and RF, while ulinastatin can protect endothelial cells and mitigate sepsis-induced microvascular barrier dysfunction by inhibiting autophagy and inflammation (Li et al., 2022). Wei et al. (2022) demonstrated that CD137 activation in LECs and macrophages from a unilateral ureteral obstruction (UUO) mouse model and IgA nephropathy (IgAN) patients induced LEC autophagy via the PI3K/AKT/mTOR pathway. This enhanced autophagy promoted LEC proliferation, migration, and tubulogenesis, contributing to fibrosis. Thus, the excessive lymphatic endothelial autophagy observed in this study has detrimental effects. Autophagy significantly modulates the function of kidney endothelial cells, highlighting the importance of timely intervention in autophagy regulation. However, the precise signaling pathways and molecular mechanisms regulating autophagy may differ among EC subtypes, warranting further investigation (Figure 3; Table 2).

**TABLE 2:** Factors influencing EC autophagy changes in RF.

Model	Autophagy-targeted cell	Drug/agent	Effect on autophagy	Mechanism and effect	References
HD-induced mice	GECs	H2S	↓ Autophagy	↑ STRAD/MO25/LKB1, ↑ P-AMPK	Kundu et al. (2013)
				↓ GEC injury and dysfunction and ECM deposition	
LPS-induced AKI mice	VECs	SA-PEG-DXM/DXM	↓ Autophagy	↓ Beclin-1, Atg5-Atg12, and ROS	Hu et al. (2017)
				↓ VEC injury, apoptosis, inflammatory, and microvascular barrier	
IgAN patients	LECs	Atg5 and Atg7	↓ Autophagy	↓ CD137/CD137L and ↓ PI3K/AKT/mTOR	Wei et al. (2022)
UUO mice		Knockdown		↓ LEC migration, tube formation, and renal fibrosis	

### 2.3 Glomerular mesangial cell autophagy and RF

**FIGURE 4:**
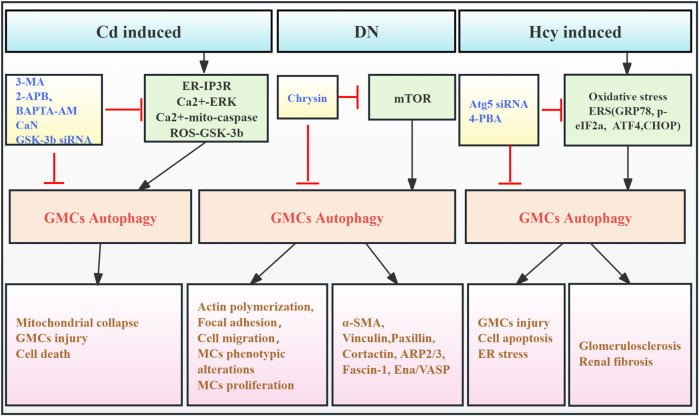
Role of GMC autophagy in RF. Agents shown in black font exacerbate renal injury by promoting podocyte autophagy, whereas agents shown in blue font protect the renal tissue by inhibiting autophagy and its associated regulators. Black arrows indicate promotion, and red “T”-shaped bars indicate inhibition. These studies demonstrate the deleterious effects of GMC autophagy activation on the kidney. GMCs, glomerular mesangial cells; MCs, mesangial cells; 3-MA, 3-methyladenine; 2-APB, 2-aminoethoxydiphenylborate; BAPTA-AM,1,2-bis (2-amino-phenoxy) ethane N,N,N,N-tetraacetic acid; CaN, calcineurin; GSK-3β, glycogen synthase kinase-3β; siRNAs, small interfering RNAs; ER, endoplasmic reticulum; ERS, endoplasmic reticulum stress; IP3R, inositol-1,4,5-tri-phosphate receptor; ERK, extracellular signal-regulated kinase; Mito, mitochondria; ROS, reactive oxygen species; mTOR, mammalian target of rapamycin; 4-PBA, 4-phenylbutyric acid; α-SMA, α-smooth muscle actin; Hcy, homocysteine; CHOP, C/EBP homologous protein.

Glomerular mesangial cells (GMCs), a critical component of the glomerular tuft, have a significant effect in regulating the glomerular filtration rate, in concert with neighboring podocytes and GECs. In RF, GMCs contribute to glomerular ECM accumulation and pro-fibrotic cytokine release (Zhao, 2019). Early studies showed that cadmium (Cd) exposure activated ERK-mediated autophagy and mitochondrial-caspase-mediated apoptosis in GMCs. Pharmacological autophagy inhibition increased GMC viability, suggesting a link between autophagy activation and Cd-induced GMC death (Wang et al., 2008). Further study revealed that Cd partially activates GMC autophagy via increased reactive oxygen species (ROS) and glycogen synthase kinase-3β (GSK-3β) (Wang et al., 2009). Additionally, one study had shown that advanced glycation end products (AGEs) upregulated autophagy-related factors (LC3 I/II, beclin-1, Atg3, and Atg7) in GMCs from diabetic kidneys. Chrysin may inhibit RF by modulating autophagy and mTOR signaling, thereby reducing diabetes-associated actin polymerization and mesangial cell motility (Lee et al., 2019). Furthermore, homocysteine (Hcy) exposure in human mesangial cells (HMCs) induces endoplasmic reticulum stress (ERS), triggering Atg5-dependent autophagy. This leads to increased Bax and caspase-3 expression, resulting in glomerular dysfunction and sclerosis. Atg5 silencing reverses these effects. These findings indicate that Hcy-induced GMC autophagy is detrimental (Liang et al., 2019). In conclusion, evidence suggests that excessive mesangial cell autophagy is associated with adverse outcomes in RF. However, research is limited, primarily focusing on early *in vitro* studies, with limited investigation into the effects of mesangial cell autophagy on RF observed in the past 5 years. Therefore, developing animal models specifically targeting GMC autophagy-related genes is crucial for advancing our understanding (Figure 4; Table 3).

**TABLE 3:** Factors influencing MC autophagy changes in RF.

Model	Autophagy-targeted cell	Drug/agent	Effect on autophagy	Mechanism and effect	References
Cd-induced MES-13 cells	GMCs	3-MA, 2-APB, CaN, and BAPTA-AM GSK-3b siRNA	↓ Autophagy	↓ ER-IP3R, Ca2+-ERK, and Ca2+-mito-caspase ↓ ROS-GSK-3b ↓ Mitochondrial collapse, GMC injury, and death	Wang et al. (2008), Wang et al. (2009)
db/db mice AGE-exposed HRMC	GMCs	Chrysin	↓ Autophagy	↓ mTOR and F-actin, cortactin, and fascin-1 ↓ GMC damage and the progression of DN.	Lee et al. (2019)
Hcy-induced HRMC	GMCs	Atg5 siRNA 4-PBA	↓ Autophagy	↓ Oxidative stress, ERS (GRP78, p-eIF2a, ATF4, and CHOP) ↓ GMC injury, glomerulosclerosis, and renal fibrosis	Liang et al. (2019)

### 2.4 Renal tubular epithelial cell autophagy and RF

Renal tubular epithelial cells (RTECs) are essential components of the renal tubules and are highly susceptible to various forms of injury, including hypoxia, toxins, and proteinuria (Liu et al., 2018). Dysregulation of signaling pathways associated with RTEC loss is a key factor in RF development (Grgic et al., 2012). While basal autophagy levels in RTECs are typically lower than in other renal cells, RTEC autophagy is often significantly upregulated during RF induced by diverse etiologies (Dai et al., 2022). Miller and Palade (1964), who used electron microscopy, were the first to observe autolytic vacuoles containing mitochondria in RTECs from a hemoglobinuria mouse model. These vacuoles demonstrated the capacity for phagocytosis. Commonly used animal models for RF include toxins or drug-induced, gene knockout, and surgical models, such as unilateral ureteral obstruction (UUO). The UUO model is most frequently employed for RF studies (Nogueira et al., 2017).

Numerous early studies using the UUO model have shown that autophagy promotes cell apoptosis (Li et al., 2010; Koesters et al., 2010; Forbes et al., 2011; Xu et al., 2013). Moreover, genetic knockout of Atg7 or Atg5 in UUO rat models inhibited autophagy, suppressing cell death, potentially interstitial inflammation and pro-fibrotic factor expression, and modulating the immune response (Livingston et al., 2016). Upregulation of beclin-1, an autophagy-related factor, is implicated in lipid droplet accumulation, renal lipotoxicity, and renal interstitial fibrosis (RIF) in the UUO model (Yan et al., 2018). Autophagy activation in the UUO model is regulated by multiple pathways, including the upregulation of C/EBP homologous protein (CHOP) (Noh et al., 2018), hypoxia-inducible factor 1-alpha (HIF-1α)/Bcl-2/adenovirus E1B 19-kDa interacting protein 3 (BNIP3) (Liu et al., 2024), AMPK/mTOR (Liu et al., 2020), p38/ERK MAPKs (Liu et al., 2020; Li K. et al., 2024), TGF-β1/Smad (Tian et al., 2020; Li K. et al., 2024), and nuclear factor kappa-light-chain-enhancer of activated B cells (NF-κB) (Tian et al., 2020), all of which contribute to RTEC autophagy and RF progression.

In an adenine-induced renal injury model, one study showed that rhein suppressed RTEC autophagy by targeting the AMPK/mTOR and p38/ERK MAPK pathways, resulting in RIF (Tu et al., 2017). Furthermore, another study demonstrated that ASIV inhibited RTEC autophagy by increasing aldehyde dehydrogenase 2 (ALDH2) expression, thereby suppressing EMT and G2/M arrest-related protein expressions and significantly reducing RF (Li et al., 2023).

In diabetic models, upregulation of Lys63-ubiquitination (Pontrelli et al., 2018) and ROS/ERK signaling (Deng et al., 2021) promotes autophagy initiation and fibrosis. Furthermore, in streptozotocin (STZ)-induced diabetic rats, H2S improved renal tissue function by modulating oxidative stress signaling, increasing superoxide dismutase (SOD) expression, and decreasing TGF-β1, matrix metalloproteinase (MMP)/tissue inhibitors of metalloproteinase (TIMP), NF-κB, and AKT signaling, thereby inhibiting aberrant RTEC autophagy (Li et al., 2017). In a palmitic acid (PA)-induced model of insulin-resistant diabetic nephropathy (DN), prostaglandin E1 (PGE1) ameliorated AKI by upregulating autophagy-mediated fibroblast growth factor 21 (FGF21), inhibiting excessive autophagy, and attenuating insulin resistance (Wei et al., 2018). Moreover, acteoside (ACT) increased transcription factor EB (TFEB) protein expression, significantly inhibited autophagy, reduced oxidative stress, and mitigated RF in DN models (Zhou et al., 2024).

In hyperuricemic nephropathy (HN), 3-methyladenine (3-MA) attenuates inflammation and NF-κB/signal transducer and activator of transcription 3 (STAT3) signaling, inhibits autophagy, downregulates the Snail and Slug transcription factors, and subsequently reduces Drp1, F-actin, and cofilin protein levels. This inhibition of mitochondrial fission reduces tubular cell G2/M cell cycle arrest, inflammation, and apoptosis and mitigates EMT and ECM accumulation. These findings reveal a vicious cycle, in which sustained uric acid injury induces mitochondrial fission and autophagy in RTECs, leading to cell damage and, ultimately, initiating necrosis or apoptosis (Hu et al., 2022; Shi et al., 2020).

Studies using the ischemia–reperfusion (I/R) model have shown that aberrant autophagy activation contributes to cell senescence, endoplasmic reticulum stress, inflammation, renal tubular cell death, and G2/M cell cycle arrest (Baisantry et al., 2016; Shu et al., 2018; Canaud et al., 2019). CG1 activation leads to the formation of rapamycin (TOR)–autophagy spatial coupling chambers (TASCCs), which induce pro-fibrotic factor release, promoting progression from AKI to CKD (Canaud et al., 2019). FXR agonists reduced autophagy and apoptosis in FXR-deficient mice subjected to I/R, mitigating early kidney injury and preventing AKI–CKD progression (Kim et al., 2021). Additionally, a study revealed that autophagy activation in the I/R model promotes the accumulation of SQSTM1/p62 and phosphorylation and activation of MAPK/ERK, initiating the downstream signal EGR1. Furthermore, in the I/R model, autophagy activation promotes SQSTM1/p62 accumulation, MAPK/ERK phosphorylation and activation, and subsequent EGR1 signaling, leading to FGF2 production and secretion, renal fibroblast activation, and, ultimately, RF. Atg7 gene knockout reversed these effects (Livingston et al., 2024).

Cystinosis, an inherited disorder caused by cystinosin (CTNS) gene mutations, frequently affects the kidneys, leading to Fanconi syndrome and RF. Studies have detected increased autophagy in the urine of cystinosis patients and in RTECs of CTNS knockout mice, suggesting a role for autophagy in promoting apoptosis (Sansanwal et al., 2010; Krohn et al., 2022). Another study showed that clusterin expression in cystinosis kidneys is associated with the co-expression of autophagy-related proteins (p62 and LC3) and apoptosis-related proteins (apoptosis-inducing factor and cleaved caspase-3). Silencing clusterin improved mitophagy in RTECs, reduced apoptosis, and mitigated renal cell damage and fibrosis (Sansanwal et al., 2015).

**FIGURE 5:**
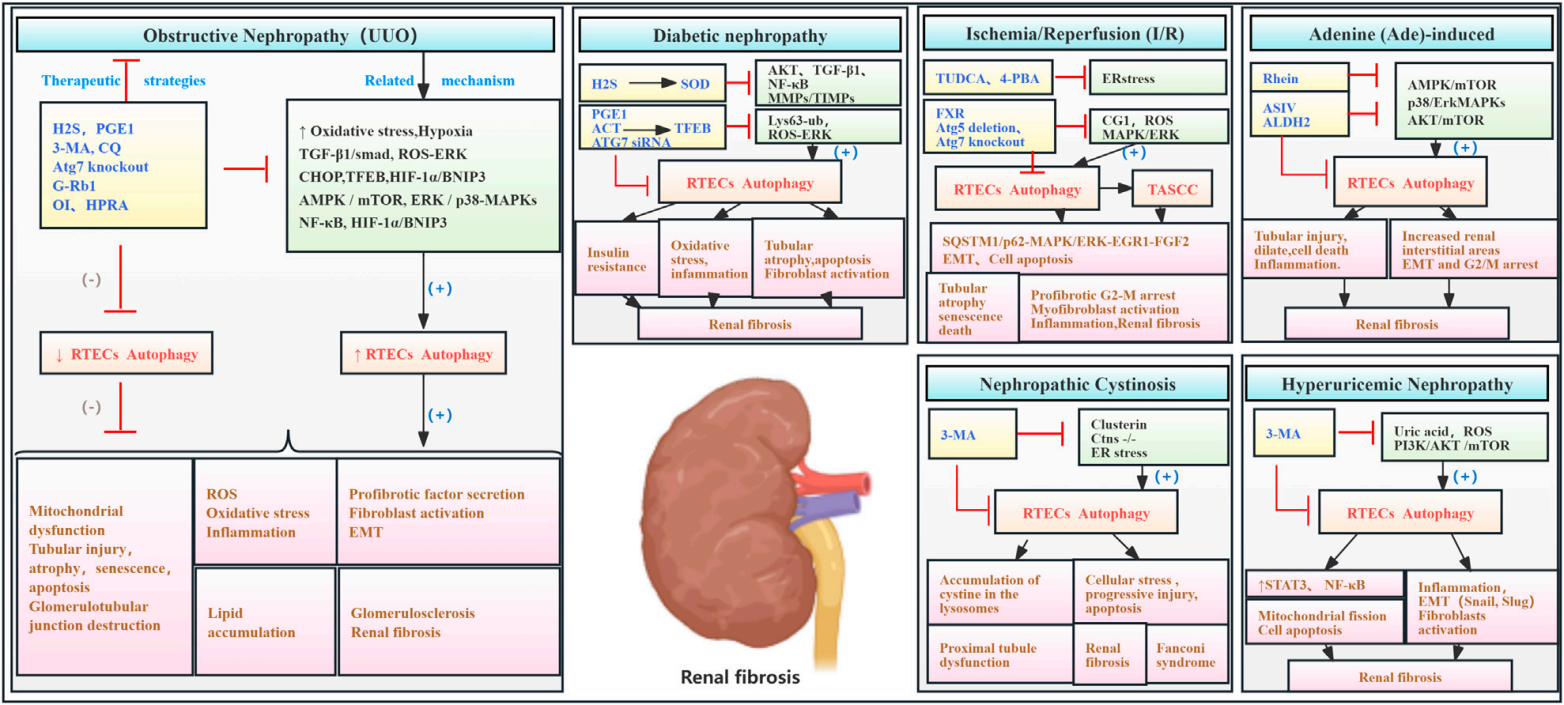
Role of RTEC autophagy in RF. Agents shown in black font exacerbate renal injury by promoting podocyte autophagy, whereas agents shown in blue font protect the renal tissue by inhibiting autophagy and its associated regulators. Black arrows indicate promotion, and red “T”-shaped bars indicate inhibition. These studies demonstrate the deleterious effects of RTEC autophagy activation on the kidney. 3-MA, 3-methyladenine; CQ, chloroquine; siRNAs, small interfering RNAs; ERK, extracellular signal regulated kinase; mTOR, mammalian target of rapamycin; 4-PBA, 4-phenylbutyric acid; CHOP, C/EBP homologous protein; FGF2, fibroblast growth factor 2; TUDCA, tauroursodeoxycholic acid; EGR1, early growth response 1; TASCCs, TOR–autophagy spatial coupling compartments; TFEB, transcription factor EB; FXR, farnesoid X receptor; ROS, reactive oxygen species; ASIV, astragaloside IV; MAPK, mitogen-activated protein kinase; HIF-1, hypoxia-inducible factors 1; ACT, acteoside; HPRA, herb pair of rhubarb–Astragalus; G-Rb1, ginsenoside Rb1; OI, 4-octyl itaconate.

Collectively, substantial evidence indicates that aberrant activation of RTEC autophagy plays a significant role in RF pathogenesis. This aberrant autophagy contributes to oxidative stress, inflammation, pro-fibrotic factor secretion, cellular senescence, apoptosis, cell cycle arrest, lipid droplet accumulation, and other changes that damage RTECs and promote fibrosis (Figure 5; Table 4).

**TABLE 4:** Factors influencing RTEC autophagy changes in RF.

Model	Autophagy-targeted cell	Agent	Effect on autophagy	Mechanism and effect	References
Chop^+/+−/−^	RTECs	Chop-deficient	↓ Autophagy	↓ Beclin 1, LC3-II, Fis1, Bax, Bcl-2, and c-caspase 3; ↑ α-TAT1	Noh et al. (2018)
UUO mice				↓ Apoptosis and mitochondrial dysfunction and renal fibrosis	
UUO mice	RTECs	HIF-1α- knockout	↓ Autophagy	↓ BNIP3	Liu et al. (2024)
Hypoxia-induced MPTC				↓ RTEC injury and renal fibrosis	
UUO mice	RTECs	G-Rb1	↓ Autophagy	↓ AMPK/mTOR and ERK/p38-MAPKs	Liu et al. (2020)
HBSS-induced HK-2				↓ RTEC injury and renal fibrosis	
UUO SD-rats	RTECs	HPRA	↓ Autophagy	↓ p38-MAPK/TGF-β1 and p38-MAPK/smad2/3 pathways	Li K. et al. (2024)
TGF-β1-induced HK-2				↑ Protect renal function and ↓ the progression of RIF	
UUO and adenine- rats; TGF-β1-induced HK-2	RTECs	OI	↓ Autophagy	↓ TGF-β/Smad and NF-κB pathways ↓ Reactive oxygen species, inflammatory, and renal fibrosis	Tian et al. (2020)
Adenine-induced rats	RTECs	ASIV	↓ Autophagy	↑ ALDH2, ↓ AKT/mTOR pathways	Li et al. (2023)
TGF-β1-induced HK-2				↓ EMT and renal fibrosis	
DN patients	RTECs	Lys63-ub	↑ Autophagy	↑ Beclin-1, LC3, p62, and caspase-3	Pontrelli et al. (2018)
HG-induced HK-2				↑ Tubular damage and the progression of DN	
STZ-induced, unilateral nephrectomy rats DN model HK-2	RTECs	ACT	↓ Autophagy	↑ TFEB; ↓ ROS, and MDA ↓ LC3-II/LC3-I and P62 ↓ RTEC injury and renal interstitial fibrosis in DN	Zhou et al. (2024)
HN SD rats	RTECs	3-MA	↓ Autophagy	↓ ROS and PI3K/AKT/mTOR ↓ NF-κβ/STAT3 and EMT (snail and slug) ↓ Inflammation and renal fibrosis	Hu et al. (2022)
IR injury mice TGF-β1-induced BUMPT	RTECs	Atg7 knockout	↓ Autophagy	↓ SQSTM1/p62-MAPK/ERK-EGR1-FGF2 ↓ EMT, cell apoptosis, and myofibroblast activation ↓ Inflammation and renal fibrosis	Livingston et al. (2024)
Cystinosis patients CDME-induced RTECs	RTECs	Ctns −/−Clusterin	↑ Autophagy	↑ LC3, LAMP2, and ROS, ↓ ATP generation ↑ Accumulation of cystine in the lysosomes, RPTE injury, renal fibrosis, and Fanconi syndrome	Sansanwal et al. (2010), Krohn et al. (2022), Sansanwal et al. (2015)

### 2.5 Fibroblast autophagy and RF

**FIGURE 6:**
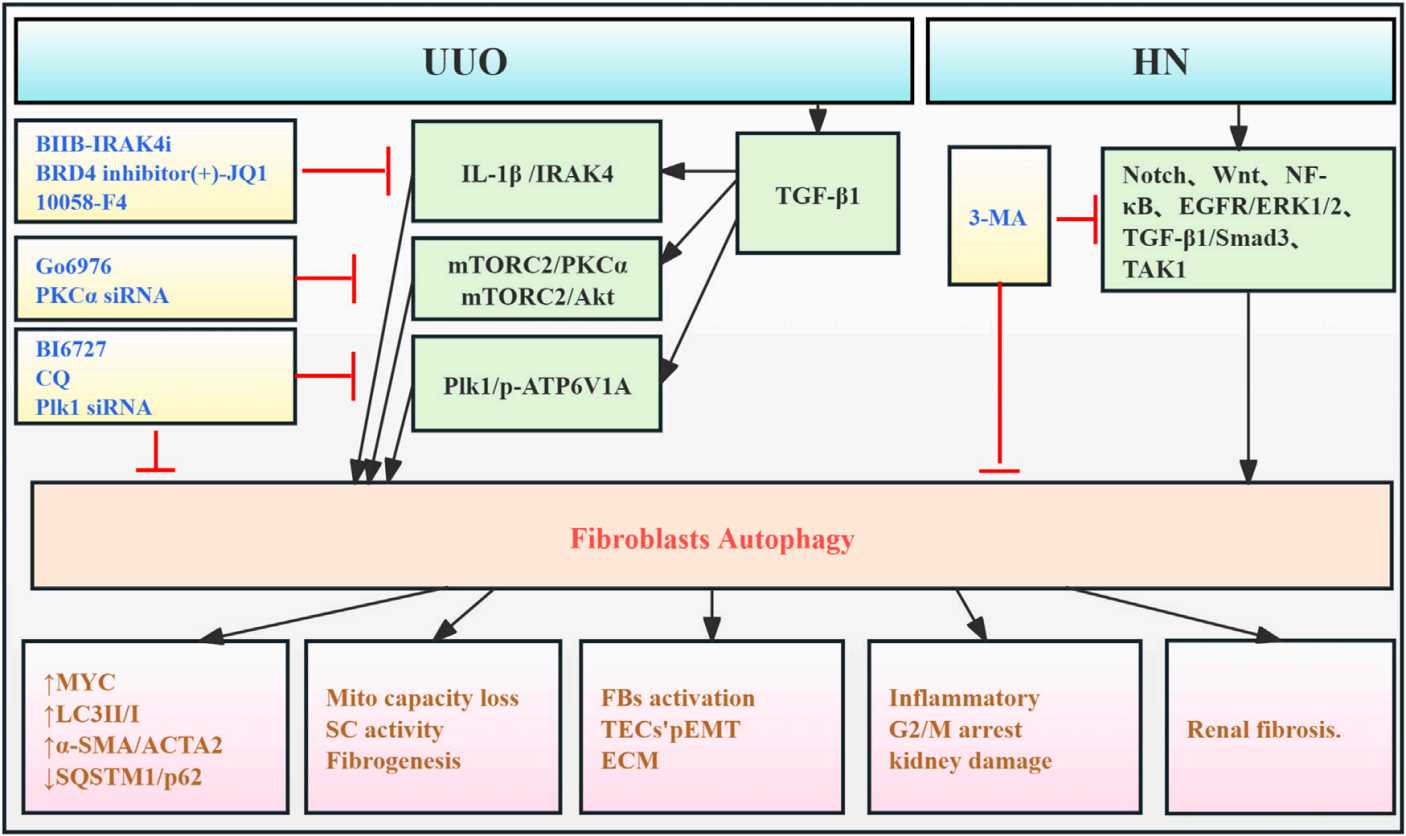
Role of FB autophagy in RF. Agents shown in black font exacerbate renal injury by promoting podocyte autophagy, whereas agents shown in blue font protect renal tissue by inhibiting autophagy and its associated regulators. Black arrows indicate promotion, and red “T”-shaped bars indicate inhibition. These studies demonstrate the deleterious effects of FB autophagy activation on the kidney. IL-1β, interleukin-1β; IRAK4, IL-1 receptor-associated kinase 4; 10058-F4, the MYC/MAX inhibitor; BIIB-IRAK4i, the IRAK4 small-molecule inhibitor; Mito, mitochondria; SCs, kidney stromal cells; KFB, kidney fibroblast; PKCα, protein kinase Cα; mTORC2, mTOR complex 2; Plk1, polo-like kinase 1; pEMT, partial epithelial–mesenchymal transition; ECM, extracellular matrix; TECs, tubular epithelial cells; BI6727, Plk1 inhibitor; CQ, chloroquine diphosphate; siRNAs, small interfering RNAs; p-ATP6V1A, ATP6V1A phosphorylation.

Renal fibroblasts (FBs), also termed stromal cells (SCs), are key components of the renal interstitium. They contribute to maintaining the renal tissue architecture and homeostasis. Under pathological conditions, these cells become activated, differentiating into myofibroblasts, which are the primary source of renal interstitial collagen deposition (Eddy, 2014). One study demonstrates that intracellular IL-1β in SCs promotes renal fibrosis. IL-1β stimulation induces autophagy, as evidenced by increased LC3-II/I and decreased SQSTM1/p62, resulting in reduced mitochondrial mass and increased MYC protein levels. This triggers MYC-dependent glycolytic proliferation, contributing to fibrosis (Lemos et al., 2018). Xue et al. (2018) reported that protein kinase C promotes fibroblast proliferation and RF by enhancing fibroblast autophagy and mTORC2/Akt signaling. Furthermore, a study observed autophagy activation in the HN mouse model and *in vitro* uric acid-induced FBs, which was associated with the upregulation of Notch, Wnt, NF-κB, EGFR/ERK1/2, TGF-β1/Smad3, and TAK1 signaling pathways, contributing to tubular damage, inflammation, G2/M cell cycle arrest, and ECM production. 3-MA, an autophagy inhibitor, significantly suppresses these pathways, reducing these cellular responses above, thereby inhibiting HN development (Bao et al., 2018). Polo-like kinase 1 (Plk1) is a key regulator of the G2/M cell cycle. Du et al. (2023) found that Plk1 was activated in fibroblasts of the proximal tubules and tubulointerstitium in CKD patients and UUO mice. This activation enhanced autophagy and regulated ATP6V1A phosphorylation, maintaining lysosomal pH. This contributed to fibroblast activation and partial epithelial-to-mesenchymal transition (EMT) in tubular cells, thereby promoting disease progression (Figure 6; Table 5).

**TABLE 5:** Factors influencing FB autophagy changes in RF.

Model	Autophagy-targeted cell	Drug/agent	Effect on autophagy	Mechanism and effect	References
UUO mice	Fibroblasts	IL-1β	↑ Autophagy	↑ LC3Ⅱ/Ⅰ and ↓ SQSTM1/p62, to ↑ MYC	Lemos et al. (2018)
IRI mice				↑ Glycolytic proliferation program and renal fibrosis	
UUO mice	Fibroblasts	PKCα siRNA	↓ Autophagy	↓ mTORC2/PKCα, mTORC2/Akt	Xue et al. (2018)
TGF-β1-induced NRK-49F				↓ Fibroblast activation and renal fibrosis	
HN rats	Fibroblasts	3-MA	↓ Autophagy	↓ Notch, Wnt, NF-κB, EGFR/ERK1/2, TGF-β1/Smad3, and TAK1	Bao et al. (2018)
UA-induced NRK-49F				↓ Fibroblast activation and development of HN	
CKD patients UUO mice TGF-β1-induced NRK-49F	Fibroblasts	Plk1 siRNA	↓ Autophagy	↓ Plk1/p-ATP6V1A and pEMT ↓ Fibroblast activation and renal fibrosis	Du et al. (2023)

### 2.6 Immune cell autophagy and RF

**FIGURE 7:**
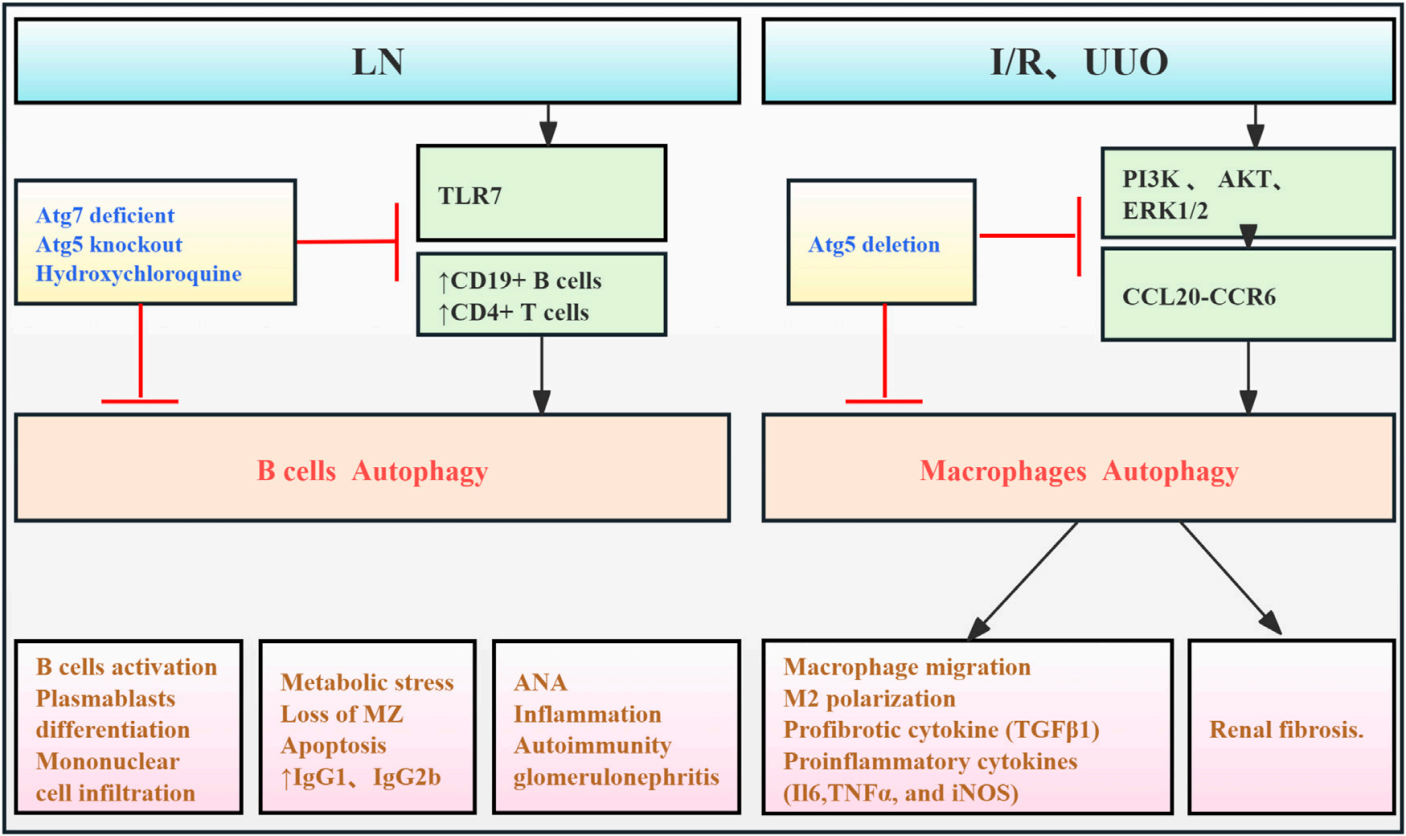
Role of immune cell autophagy in RF. Agents shown in black font exacerbate renal injury by promoting podocyte autophagy, whereas agents shown in blue font protect the renal tissue by inhibiting autophagy and its associated regulators. Black arrows indicate promotion, and red “T”-shaped bars indicate inhibition. These studies demonstrate the deleterious effects of immune cell autophagy activation on the kidney. ANA, antinuclear antibodies; Toll-like receptor (TLR) 7; MZ, marginal zone; I/R, ischemia/reperfusion; CCL20, chemokine (C–C motif) ligand 20; Atg, autophagy-related; PI3K, phosphoinositide-3-kinase regulatory subunit 1; UUO, unilateral ureteral obstruction; ERK, extracellular signal-regulated protein kinase, AKT, thymoma viral proto-oncogene 1; LN, lupus nephropathy.

Literature review indicates that autophagy is involved in both renal parenchymal cell injury and the modulation of immune responses in kidney diseases (Leventhal et al., 2014). Enhanced autophagy in B cells from mice and humans with lupus induces cellular stress. This suggests that autophagy is crucial for the survival and differentiation of autoreactive B cells, especially during early development and plasmablast formation. Therefore, targeting autophagy may represent a novel therapeutic strategy for SLE (Clarke et al., 2015). Weindel et al. (2015) demonstrated that autophagosomes in B cells contribute to SLE pathogenesis by enhancing the delivery of self-antigens, including endogenous retroviral elements and cytoplasmic RNA from internalized immune complexes, to TLR7 within endosomes. In Tlr7.1Tg Atg5 knockout mice, blocking B-cell autophagy restored the marginal zone (MZ), reduced inflammatory cytokine levels, and reduced renal injury and fibrosis. Zhu et al. (2024) demonstrated the critical role of macrophage autophagy in renal macrophage migration and fibrosis. Using myeloid cell-specific Atg5 knockout mice, they showed that blocking autophagy disrupted the CCL20–CCR6 axis, inhibiting macrophage migration and M2 polarization, and thus improving RF in renal I/R and UUO models. Collectively, these findings indicate that immune cell autophagy contributes to enhanced inflammatory responses and fibrosis through increased cellular stress, proliferation, and migration (Figure 7; Table 6).

**TABLE 6:** Factors influencing immune cell autophagy changes in RF.

Model	Autophagy-targeted cell	Drug/agent	Effect on autophagy	Mechanism and effect	References
LN patients	B cells	Atg7 deletion	↓ Autophagy	↓ CD19^+^ B and CD4^+^ T cells, ↓ Bcl-2	Clarke et al. (2015)
NZB/W mice				↓ Apoptosis, autoimmunity, and glomerulonephritis	
Tlr7.1 Tg and Tlr7.1 Tg Atg5 KO mice	B cells	Atg5 deletion	↓ Autophagy	↓ TLR7	Weindel et al. (2015)
				↓ Autoimmunity, inflammation, anemia, and glomerulonephritis	
I/R mice	Macrophage	Atg5 deletion	↓ Autophagy	↓ PI3K, AKT, ERK1/2, and CCL20-CCR6	Zhu et al. (2024)
UUO mice				↓ Macrophage migration, inflammation, and fibrosis	

## 3 The potential value of autophagy in RF

### 3.1 Update and application of the detection methods of autophagy in renal diseases

Early-stage renal disease often lacks overt clinical symptoms, and traditional biomarkers such as serum creatinine (Cr), blood urea nitrogen (BUN), and glomerular filtration rate (GFR) have limited sensitivity, delaying diagnosis and optimal treatment (Ho et al., 2013). Early detection of key biomarkers facilitates timely intervention and improved patient outcomes. Recent advances in understanding autophagy in RF have significantly improved the detection methods and diagnostic capabilities. Established autophagy detection methods, including transmission electron microscopy, fluorescence microscopy, Western blotting, and quantitative PCR, are now being integrated with “omics” technologies (metabolomics, proteomics, and transcriptomics) and bioinformatics to provide a more comprehensive understanding of autophagy and its regulation in RF. For example, Rinschen et al. (2022) integrated phosphoproteomics, proteomics, metabolomics, and super-resolution imaging to analyze PTC-induced VPS34 deficiency in mice. Their findings demonstrated that PI3K/VPS34 regulated autophagy and enzymatic function in renal proximal tubule cells (PTCs). Zhao et al. (2023) performed transcriptomic and proteomic analyses of glomeruli from 25 controls and 50 DN patients. Pathway enrichment analysis revealed APA gene enrichment in inflammation-related processes, including ERS, NF-κB signaling, and autophagy, suggesting a role for APA in DN progression. Huang et al. revealed that proteomic analysis of human renal tissue downregulated VPS37 and ATG4B and upregulated NBR1 expression, which are associated with autophagy inhibition, suggesting that these proteins may serve as sensitive serum biomarkers for early DN detection and that autophagy inhibition may be a therapeutic target (Huang et al., 2022). In conclusion, autophagy and its regulatory factors show promise as early diagnostic biomarkers for kidney disease, and improved detection methods may enhance early diagnosis and the detection of RF progression.

### 3.2 Regulation of autophagy as a serviceable therapeutic strategy for CKD

Progression of CKD to ESKD is often accompanied by severe RF, necessitating dialysis or transplantation, which carries substantial economic and physical burdens for patients (Turner et al., 2012). RF is a critical pathological hallmark of CKD, yet targeted therapies remain largely in the preclinical stage (Ruiz-Ortega et al., 2022). While glucocorticoids are commonly used to treat CKD, their efficacy is limited, and they can cause numerous adverse effects, including hypertension, infections, and metabolic disorders (Ponticelli and Locatelli, 2018). Identifying novel therapeutic targets and early intervention strategies is crucial to prevent irreversible RF. Sansanwal et al. (2010) identified autophagy as a potential therapeutic target for nephrotic kidney injury, observing autophagy abnormalities in FBs and RTECs from cystinosis patients.

Renal biopsies from patients with AKI exhibit increased RTEC autophagy and elevated FGF2 expression, suggesting a link between autophagy and RF (Livingston et al., 2023). Therefore, inhibiting autophagy is a potential strategy for mitigating kidney disease progression. Preclinical studies have investigated autophagy inhibitors (e.g., 3-MA (Livingston et al., 2016; Shi et al., 2020) and chloroquine (Livingston et al., 2016; Deng et al., 2021)) or autophagy gene silencing (e.g., atg7 (Livingston et al., 2016; Livingston et al., 2024; Livingston et al., 2023) and atg5 (Baisantry et al., 2016)) to suppress autophagy activation, potentially improving renal function and mitigating fibrosis. Research into the molecular mechanisms regulating autophagy has yielded promising results. Deletion of VPS34 induces autophagy activation, potentially mitigating glomerulosclerosis (Chen et al., 2013). Hu et al. (2017) designed SA-PEG-DXM/DXM micelles to inhibit LPS-induced autophagy in endothelial cells, potentially improving AKI. Silencing clusterin reduces autophagy and may represent a therapeutic target for cystinosis nephropathy (Sansanwal et al., 2015). Canaud et al. (2019) identified TASCC accumulation as a key factor contributing to RF in an IRI mouse model. Plk1 promotes autophagy in fibroblasts, suggesting it as a potential therapeutic target (Du et al., 2023). VEGF-C promotes CD137L secretion, initiating autophagy in lymphatic endothelial cells and contributing to RIF (Wei et al., 2022). These findings highlight the potential of these molecular pathways as therapeutic targets for RF by modulating autophagy.

Traditional Chinese medicine (TCM) and other natural plant compounds offer advantages such as favorable safety profiles, widespread availability, and affordability. These natural compounds show promise in CKD treatment research and represent a valuable resource for developing novel therapeutics. For example, MFSD, a traditional Chinese medicine formula, contains aconitum, poplar, ginger, Ephedra, licorice, and Atractylodes. These ingredients have a long history of use in treating kidney disease. Studies suggest that MFSD reduces autophagy by downregulating Wnt/β-catenin signaling, potentially mitigating podocyte damage in MN and delaying disease progression (Gao et al., 2022). Furthermore, numerous agents have been shown to improve renal function and reduce RF by reducing autophagy in various models, including G-Rb1 (Liu et al., 2020), OI (Tian et al., 2020), and HPRA (Li J. et al., 2024) in the UUO model; rhein (Tu et al., 2017) and ASIV (Li et al., 2023) in the adenine-induced renal injury model; chrysin (Lee et al., 2019), ACT (Zhou et al., 2024), H2S (Li et al., 2017), and PGE1 (Wei et al., 2018) in the DN model; FXR (Kim et al., 2021) in the I/R model; and ulinastatin (Li et al., 2022) in sepsis models. These findings suggest that modulating autophagy represents a promising therapeutic strategy for kidney diseases.

## 4 Limitations of autophagy and the problems it faces

Despite preclinical and clinical evidence implicating autophagy in maladaptive renal repair, translating autophagy-modulating therapies for RF faces several challenges. First, this review shows that prolonged autophagy activation in renal cells impairs tissue repair. This underscores the need for precise control over autophagy duration, activation level, and treatment timing when designing therapeutic interventions. Second, the role of autophagy in RF is complex. While basal autophagy’s protective effects on cellular homeostasis and fibrosis mitigation are well-established (Barutta et al., 2023; Guo et al., 2024; Njeim et al., 2024; Dai et al., 2022; Choi, 2020), evidence regarding the detrimental effects of sustained autophagy remains limited. Autophagy modulation outcomes are influenced by disease etiology, disease stage, and cell-type-specific molecular mechanisms. The precise role of autophagy in kidney function remains unclear. Third, while pharmacologic autophagy modulation holds therapeutic promise, currently no specific autophagy-targeting drugs exist. For example, 3-MA (Livingston et al., 2016; Shi et al., 2020) and chloroquine (Livingston et al., 2016; Deng et al., 2021) inhibit autophagy but also affect other signaling pathways, limiting their clinical utility due to potential side effects. The therapeutic potential of TCM and natural plant compounds in modulating autophagy for kidney disease remains largely unexplored. Future research should comprehensively investigate the dynamic regulation of autophagy across diverse CKD etiologies to validate and refine these findings. Understanding autophagy’s molecular mechanisms in RF is crucial for elucidating maladaptive kidney repair and developing novel strategies to prevent CKD progression.

## 5 Conclusion

CKD presents a significant global health challenge, hampered by subtle early symptoms, limited sensitivity of early detection methods, and a lack of specific therapies (Ho et al., 2013; Turner et al., 2012; Drawz and Rahman, 2015). This highlights the urgent need for improved early detection, diagnosis, and treatment strategies. While the role of autophagy in renal fibrosis remains a subject of ongoing investigation, with some studies suggesting a protective role, this review focuses on the substantial evidence supporting a pro-fibrotic mechanism driven by sustained, abnormal autophagy activation within intrinsic renal cells (glomeruli, tubules, and interstitium). A significant body of research currently centers on the association between RTEC autophagy and RF, highlighting the crucial role of the RTEC response in RF pathogenesis. Autophagy upregulation contributes to RF by promoting oxidative stress, inflammation, pro-fibrotic factor secretion, cellular senescence, apoptosis, cell cycle arrest, and lipid accumulation, ultimately leading to renal cell damage, EMT, ECM deposition, myofibroblast activation, and fibrosis progression. Furthermore, autophagy-related factors show promise as early biomarkers for kidney disease, potentially enhancing early CKD diagnosis, although further research is required. Additionally, autophagy and its regulatory factors represent promising therapeutic targets for RF, offering potential avenues for novel drug development to treat CKD. However, research into autophagy-specific targets remains limited, and dedicated autophagy-modulating therapies are yet to be developed. This review contributes to a comprehensive understanding of the pro-fibrotic mechanisms of autophagy in renal fibrosis and aims to inform the development of effective strategies for CKD prevention and treatment.
